# Chronic active Epstein-Barr virus infection involving gastrointestinal tract mimicking inflammatory bowel disease

**DOI:** 10.1186/s12876-020-01395-9

**Published:** 2020-08-05

**Authors:** Weijia Xu, Xiaoyun Jiang, Jiajie Chen, Qiqi Mao, Xianguang Zhao, Xu Sun, Liang Zhong, Lan Rong

**Affiliations:** 1 Department of Gastroenterology, Huashan Hospital (North), Fudan University, Shanghai, China; 2 Department of Gastroenterology, Huashan Hospital, Fudan University, Shanghai, China

**Keywords:** Inflammatory bowel disease, Epstein-Barr virus, Chronic active Epstein-Barr virus infection, Differential diagnosis

## Abstract

**Background:**

Chronic active Epstein-Barr virus infection (CAEBV) is a rare disease, which is difficult to be differentiated from inflammatory bowel disease (IBD). To cause the attention, we present twelve cases of CAEBV in immunocompetent patients with gastrointestinal tract involvement.

**Methods:**

Twelve patients who fulfilled the diagnostic criteria of CAEBV were enrolled in this retrospective study. The control group was consisted of twenty-four IBD patients with EBV-DNA value increased in peripheral blood. The clinicopathologic and endoscopic characteristics were reviewed and analyzed.

**Results:**

The major clinical presentations of CAEBV patients were intermittent fever (100%), hepatomegaly/splenomegaly (58%), lymphadenopathy (50%), diarrhea (50%) and hematochezia (50%). Compared with IBD patients, the incidence of intermittent fever and increased level of ferritin were significantly higher among CAEBV patients. The median values for EBV detected in peripheral blood were significantly higher in CAEBV group (1.42*10^6 copies/μg) than in IBD group (3.2*10^3 copies/μg, *p*<0.05). The main endoscopic findings of CAEBV included multifocal or isolated, irregular, multiform ulcers and diffuse inflammation, lacking of typical cobblestone appearance. Ten patients died within 5 years of disease onset. The average survival time is 21 months.

**Conclusions:**

Symptoms such as intermittent fever, increased level of ferritin and atypical endoscopic findings could be a sign for CAEBV. Early detections of EBV-DNA in serum and EBV-encoded small nuclear RNA (EBER) by in situ hybridization in intestinal tissue are essential for differential diagnosis between CAEBV and IBD.

## Background

Epstein-Barr virus (EBV) infections are usually acquired during childhood or adolescence. After primary infection, Epstein-Barr virus normally establishes a permanent latent state in B lymphocytes of immunocompetent hosts [1]. In western countries, EBV usually infects B cells. While in Asia, the disease sometimes involves T or NK cells, which relates with poor prognosis [2, 3]. Under some circumstances, target cells infected by Epstein-Barr virus expand and cause persistent or recurrent symptoms. This leads to a wide range of lymphoproliferative disorders, including posttransplant lymphoproliferative disease, Hodgkin or non-Hodgkin lymphoma and chronic active Epstein-Barr virus infection (CAEBV) [4].

Horwitz et al. [5] first described cases with high IgG antibody titers against EBV-replicating antigens which manifested as persistent or intermittent high fever and lymphadenopathy. In 2005, Joan Robinson [6] reported a case with chronic active Epstein-Barr virus infection who presented with an inflammatory bowel disease (IBD) -like symptom.

Many studies have reported the presence of Epstein-Barr virus in colonic mucosa of IBD patients [7, 8], including latent without any systemic symptoms and acute but self-limited infection. Due to the similar symptoms, it is a clinical challenge to discern whether the severity of symptoms is attributed to chronic Epstein-Barr virus infection, or the exacerbation of IBD. Misdiagnosis of CAEBV and IBD may cause delay in treatment, so it is significantly important to make the accurate diagnosis at patients’ first encounter.

Chronic active Epstein-Barr virus infection with gastrointestinal tract involvement is rather rare and is often misdiagnosed. The case reports in immunocompetent individuals are sporadic. Our study retrospectively collected twelve cases of CAEBV involving gastrointestinal tract and summarized the clinical manifestations, endoscopic, pathological features and prognosis of them.

## Methods

### Patients

From 2013 June to 2019 June, twenty-five patients who were diagnosed with gastrointestinal lesions with a positive result of Epstein-Barr virus testing on colonic mucosa were reviewed. Thirteen were excluded because of malignant tumor, acute infection or enteritis without systemic symptoms. The rest twelve patients who fulfilled the diagnostic criteria of CAEBV [4, 9] were retrospectively enrolled in our study. The inclusion criteria were as followed, 1) persistent or recurring infectious mononucleosis-like symptom, including fever, hepatosplenomegaly and lymphadenopathy, 2) Unusual pattern of anti-EBV antibodies, and/or detection of high EBV-DNA load in peripheral blood or affected tissues, 3) chronic illness which cannot be explained by other known disease processes at diagnosis.

Twenty-four patients were retrospectively enrolled as control group. The inclusion criteria were as followed, 1) confirmed IBD diagnosis based on clinical, endoscopic and histological features, 2) a positive result of EBV-DNA in peripheral blood.

Clinical manifestations, demographic, laboratory, endoscopic, pathological findings and follow-up information were acquired from chart reviewing.

### Quantitation of EBV-DNA and EBER detection by in situ hybridization

Whole blood obtained from patients was centrifuged and separated into plasma and cell fractions. A quantitative real-time PCR assay was performed. The amount of EBV-DNA was calculated as viral DNA copies per milliliter blood.

Mucosa for EBV-encoded small nuclear RNA (EBER) detection was biopsied from inflamed tissues. It was fixed by formalin and embedded in paraffin blocks. In situ hybridization (ISH) was performed according to the manufacturer’s instructions with EBER ISH Kit (ZSGB-BIO, Ltd., Beijing, China). The 4-μm-thick sections were transferred to pretreated slides and stored at 37 °C overnight. Then, slides were deparaffinized with xylene for 10 min and rehydrated with anhydrous alcohol for 5 min. After being washed with distilled water and dried, slides were digested by gastric enzyme for 30 min. The slides were incubated at 37 °C overnight with hybridization solution containing the EBER-probes and then washed with phosphate buffer saline. The anti-biotin antibody was applied. The slides were then counterstained with hematoxylin, mounted, and viewed with a standard light microscope.

EBER-positive staining was recognized as a brown color seen in the nucleus of cells. A known EBV-positive nasopharyngeal carcinoma was used as a positive control.

### Statistical analysis

Baseline demographic and disease features are presented by using descriptive statistics. Continuous variables were described as median, while categorical variables were expressed as counts and percentages, with all range. Student’s t tests were used to compare continuous variables. A *P* value below 0.05 was considered statistically significant. Statistical analysis was conducted by SPSS version 16.0.

## Results

Among the twelve patients who were diagnosed CAEBV with gastrointestinal tract involvement, eleven were males and one was female with a median age of 50 (range, 24–72). None of them had a history of transplantation, HIV infection or exposure to immunosuppressants. Among control group, sixteen were males and eight were females with a median age of 45 (range, 21–70). In control group, eleven were diagnosed Crohn disease (CD), while thirteen were diagnosed ulcerative colitis (UC).

### Clinical features

The clinical features of all CAEBV patients and control group are summarized in Table 1. Gastrointestinal symptoms such as vomiting, diarrhea, abdominal pain and hematochezia were presented in all patients. The systemic symptoms were more often observed in CAEBV patients, including intermittent fever (100%), hepatomegaly/splenomegaly (58%) and lymphadenopathy (50%). Compared to control group, the incidence of intermittent fever was significantly higher among CAEBV patients (*p*<0.05).

**Table 1:** Clinical symptoms of CAEBV group and control group

	CAEBV(*n* = 12)	Control (*n* = 24)	*p*-Value
Diarrhea	6 (50%)	18 (75%)	> 0.05
Abdominal pain	4 (33%)	18 (75%)	> 0.05
Vomiting	3 (25%)	0	> 0.05
Hematochezia	6 (50%)	13 (54%)	> 0.05
Intermittent fever	12 (100%)	3 (13%)	< 0.05
Hepatomegaly/Splenomegaly	7 (58%)	5 (21%)	> 0.05
Lymphadenopathy	6 (50%)	4 (17%)	> 0.05

### Laboratorial findings

The laboratory findings of both CAEBV patients and control group are summarized in Table 2. Blood examination showed increased levels of platelet, erythrocyte sedimentation rate (ESR) and C-reactive protein (CRP) both in CAEBV patients and control group, while level of ferritin in CAEBV patients increased significantly (*p*<0.05). Positive results of Epstein-Barr virus DNA in peripheral blood were detected in all CAEBV patients (10/10, 100%. Two did not complete the test) and all control group patients (24/24, 100%). The median values for Epstein-Barr virus were significantly higher in CAEBV patients (1.42*10^6 copies/mL) compared to control group (3.2*10^3 copies/mL) (*p*<0.05). Clinical data of all enrolled CAEBV patients are summarized in Table 3.

**Table 2:** Laboratory index of CAEBV group and control group

	CAEBV (*n* = 12)	Control (*n* = 24)	*p*-Value
Increased PLT	59% (7/12)	45% (11/24)	> 0.05
Increased ESR	67% (8/12)	63% (15/24)	> 0.05
Increased CRP	100% (12/12)	45% (11/24)	> 0.05
Increased Fe protein	100% (12/12)	8% (2/24)	f 0.05

**Table 3:** Clinical characteristics of 12 CAEBV patients

NO	Sex	Age ranges	Course of disease	Symptoms	EBV-DNA (copies/ml)	Surgery	Medicine	Prognosis
1	1	20–30	3 years	Fever, hematochezia	4.34*10^6	NO	Steroids, antibiotics and antiviral-drugs	Dead
2	1	50–60	3 months	Fever, vomiting, diarrhea	NA	NO	Steroids, antibiotics and thalidomide	Dead
3	1	40–50	4 months	Fever, hematochezia	NA	NO	Steroids, antiviral-drugs and immunoglobulin	Dead
4	1	40–50	3 years	Fever, vomiting, diarrhea	4.0*10^7	NO	Steroids and immunoglobulin	Dead
5	1	30–40	2 months	Fever, hematochezia	1.14*10^7	NO	Steroids and antivirus	Dead
6	1	40–50	2 years	Fever, hematochezia	1.22*10^5	YES	Steroids	Dead
7	1	20–30	1 year	Fever, diarrhea	1.54*10^6	NO	Steroids	Dead
8	2	30–40	3 years	Fever, vomiting, abdominal pain, diarrhea	3.12*10^5	NO	Steroids, antiviral-drugs and immunoglobulin	Dead
9	1	40–50	17 months	Fever, abdominal pain	1.09*10^3	YES	Steroids, antiviral-drugs	Dead
10	1	40–50	2 months	Fever, abdominal pain, hematochezia	1.29*10^6	NO	Steroids	Survive
11	1	50–60	1 month	Fever, abdominal pain, diarrhea	1.85*10^4	NO	Steroids and hemopoietic stem cell transplantation	Survive
12	1	70–80	3 months	Fever, diarrhea, hematochezia	1.63*10^6	NO	Steroids and antibiotics	Dead

In consideration for patients’anonymity, we amend sex from “male” and “female” to “1” and “2”for publication

### Endoscopic features

**Fig. 1:**
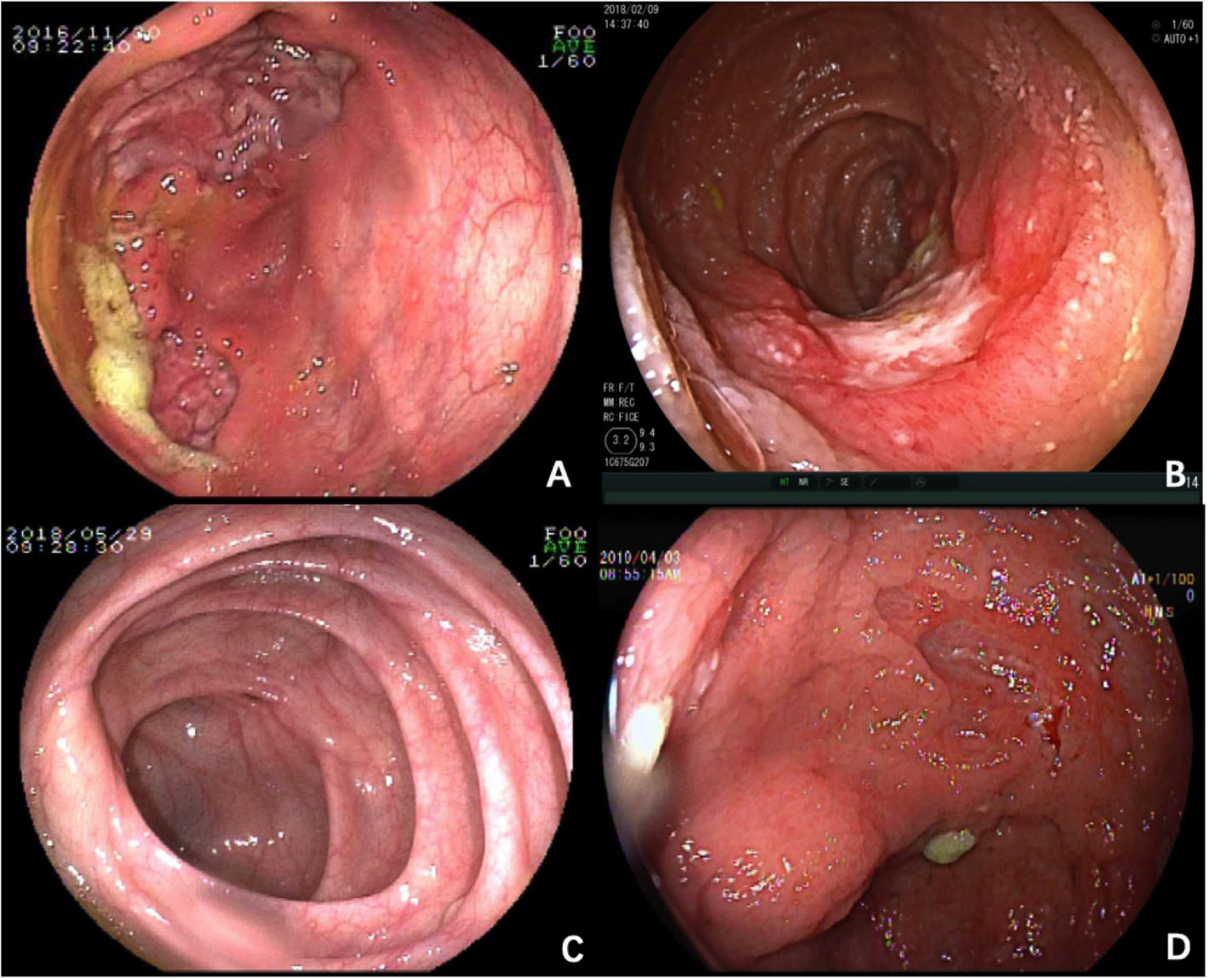
Endoscopic findings of CAEBV patients. **a** isolated giant ulcer in the ileocecum (case 2); **b** solitary longitudinal ulcer with clear rim in jejunum (case 4); **c** Diffuse inflammation in ileocecum (case 8); **d** multifocal irregular ulcers in the ascending colon (case 10)

Endoscopic manifestations of enrolled CAEBV patients are summarized in Table 4. The most frequently affected sites were colon (10/12), followed by small intestine (6/12) and stomach (1/12). Colon and small intestine were involved together in five cases. Six cases displayed profound and irregular ulcer with clear boundary, about 1.5–3.0 cm in diameter. Two cases displayed diffusely distributed, numerous shallow and small ulcers with inflammation. One case presented rigidity of intestinal wall and remarkable lymphangiectasia. One case showed a solitary longitudinal ulcer with clear boundary in jejunum. No cobblestone appearance was observed in all twelve cases (Fig. 1).

**Table 4:** Endoscopic manifestations of 12 CAEBV patients

NO	Involved location	Segmental Distribution	profound ulcer	shallow ulcer	Boundary	mucosal hyperplasia	mucosal erosion	Rigidity of intestinal wall	Stenosis
			(diameter)						
1	jejunum, terminal ileum and colon	no	no	yes	clear	no	yes	yes	no
2	ileum and colon	yes	yes (3 cm)	no	clear	yes	yes	no	no
3	ileum and colon	no	no	yes	clear	yes	yes	no	no
4	jejunum, ileum and colon	yes	yes (2 cm)	yes	clear	yes	yes	no	no
5	stomach and colon	yes	yes (2 cm)	yes	clear	yes	no	yes	no
6	ileum	yes	yes (3 cm)	yes	clear	yes	yes	no	no
7	colon	yes	yes (1 cm)	no	clear	no	yes	no	no
8	colon	yes	no	no	clear	no	no	no	no
9	ileum	yes	no	yes	clear	no	yes	no	no
10	colon	yes	yes (1.5 cm)	no	clear	yes	yes	no	no
11	colon	yes	no	yes	clear	no	no	no	no
12	colon	yes	no	yes	clear	yes	yes	no	no

### Pathological findings

**Fig. 2:**
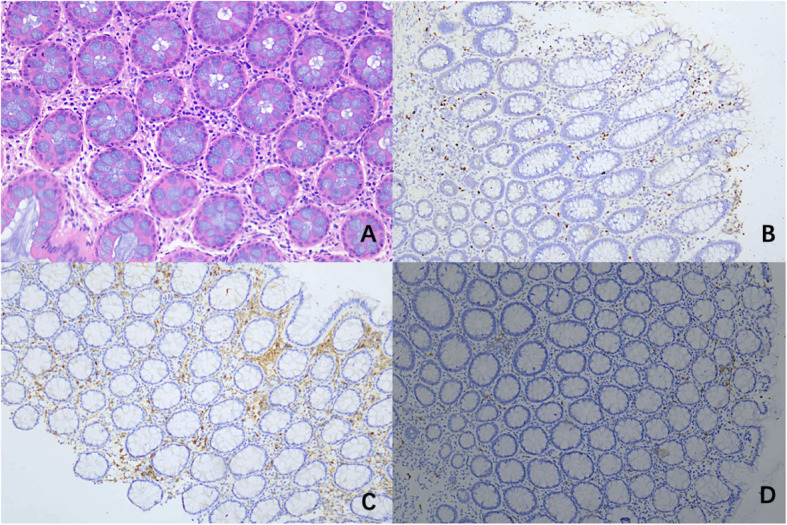
Histopathological findings of CAEBV patient (case 11). **a** Haematoxylin and eosin (H&E) staining showed Lymphoid cells distributed in muscular layer and serosa; **b**, **c** Immunohistochemical staining revealed positive expressions of CD4+ (**b**), CD20+ (**c**); (**d**) In situ hybridization indicating positive EBER

In all CAEBV patients, chronic mucositis and erosion with clusters of lymphocytes infiltrating were observed and lymphocytes infiltrate into the submucosa and muscular layer occasionally. All the infiltrating lymphocytes exhibited polyclonal proliferation instead of monoclonal proliferation. The samples subjected to immunohistochemical staining were positive or partially positive for CD3, CD4 or CD56, which were consistent with EBV-associated T cell or NK cell lymphoproliferative disease. No granulomas were observed (Fig. 2). We had two patients went through surgeries because of intestinal perforation. In both surgical specimens, transmural inflammation and aggregation of lymphocytes were observed.

The results of in situ hybridization for EBER were all positive in CAEBV patients. More EBER-positive lymphocytes were identified in surgical sample. The positive lymphocytes distributed unevenly and were mainly observed near the ulcers or lymphocyte-rich regions.

### Prognosis

Up to 2019 June, ten patients in CAEBV group died within 5 years of disease onset. The common direct cause of death included hemorrhage, disseminated intravascular coagulation and hemophagocytic syndrome. The average survival time is 21 months. One of the alive patients is under hemopoietic stem cell transplantation.

## Discussion

In this study, we report twelve cases of CAEBV with gastrointestinal tract involvement and compare the clinicopathologic characteristics of them with IBD patients who have latent EBV infection. The results reveal some similarities between CAEBV and IBD, such as gastrointestinal symptoms and increased level of ESR and CRP. Involvement of other organs, such as enlargement of liver, spleen, and lymph nodes, is more often observed in CAEBV patients. If the lesions are only located in gastrointestinal tract, the differential diagnosis could be very difficult. We identify some characteristics, including intermittent fever, extremely high level of ferritin and atypical endoscopic manifestations, which could provide some evidence to avoid misdiagnosis.

The enrolled CAEBV patients commonly presented with gastrointestinal symptoms of diarrhea and abdominal pain, clinically mimicking IBD. Some of the distinctions between clinical presentations of CAEBV and IBD are as follows: 1) The abdominal pain in CAEBV is more severe, hardly relieves spontaneously. 2) Fever, especially inexplicable intermittent high fever, is more common in CAEBV patients. 3) The level of ferritin increases drastically in CAEBV, which correlates with EBV infection.

In our study, the endoscopic features included inflammations and ulcers of variable morphological characteristics. The ulcers are irregular and multiform, which could be profound or superficial, isolated or multifocal. The signs are distinct from typical cobblestone appearance observed in CD and a uniform and continuous inflammation observed in UC [10]. Liu et al. [11] have reported some CAEBV cases with numerous shallow, small, and irregular ulcers in both colon and small intestine. These signs were observed in some of our patients. Since few articles have reported endoscopic findings in CAEBV with gastrointestinal tract involvement, our findings may provide some information for the awareness of the rare disease.

The results of EBV-DNA quantitation analysis in peripheral blood were positive in all enrolled patients, but higher in CAEBV group. The median values were 1.42*10^6 copies/mL in CAEBV, compare to 3.2*10^3 copies/mL in IBD, suggesting the disease is linked to viral replication. On the contrary, Epstein-Barr virus antibody tests are less useful because the antibody profile can mimic a latent EBV infection in IBD patient. Kimura et al. [12] have analyzed thirty CAEBV patients, and discovered not all patients had high titers of EBV-specific antibodies, but all patients had high viral loads in their peripheral blood. He recommended that viral load detected in peripheral blood mononuclear cell (PBMC) could be a criterion for disease diagnosis and an indicator of therapeutic efficacy. Yamamoto et al. [13] reported patients with CAEBV infection had cell-free EBV DNA in plasma, suggesting the presence of EBV DNA in plasma may have significance for the diagnosis of CAEBV infection. Among the twelve patients, we have two patients tested for EBV DNA in plasma, the results were 8.9*10^2 copies/mL and 6.3*10^3 copies/mL respectively. With the development of laboratory technology, more patients will be tested in PBMC and plasma. The combined application of EBV DNA tested in PBMC and plasma could be useful for CAEBV diagnosis.

At present, the golden standard for demonstrating Epstein-Barr virus infection in lesion is in situ hybridization for EBER. However, the criteria regarding percentage of EBER-positive cells for the definition of EBV infection is still not established. In the previously published articles, the threshold differs from 10 to 20% [14, 15]. Liu et al. [11] have reported eleven cases among which all surgery samples had more than 100 EBV+ cells/HPF, and the biopsy samples were more than 30 EBV+ cells/HPF. Our study showed the similar result, which is correspondent to specimen quantity. It should be noticed that we also had one case with 30 EBV+ cells/HPF in control group. As the patient had no sign of fever, hepatomegaly or other systemic symptoms and his result of EBV-DNA in peripheral blood was negative, we considered this patient clear of chronic active Epstein-Barr virus infection. Therefore, single evidence of EBER-positive is not enough for diagnosis of CAEBV. Other information, including symptoms, laboratorial results, endoscopic findings and histopathological manifestations should be combined to consider. This is consistent with previous published article [16, 17].

Chronic active Epstein-Barr virus infection often results in poor prognosis. A large cohort study [18] in Japan reported that 43% of patients died during follow-up periods that ranged from 5 months to 12 years after the onset of severe CAEBV infection. Risk factors for death include late onset of disease (onset age>8 years), thrombocytopenia and EBV infection on T cell. Successful allogeneic, hematopoietic stem cell transplantation was reported [19]. However, the transplantation constitutes a substantial risk to recipient patients. The post-transplantation mortality rate for a series of Japanese patients was 50% [18]. In our study, ten patients died within 5 years of disease onset. One of the alive patients is now under stem cell transplantation.

In 1999, Yanai et al. [8] applied in situ hybridization technique to detect the presence of EBER-positive cells in intestinal mucosa of IBD patients. Previous researches showed the data about EBV infection in colonic mucosa of IBD patients varied from 45.5 to 81.0% [16, 20–22]. Li et al. [23] recently completed a cross-sectional study in China, they found that EBV was detectable in 33 out of 99 IBD patients (33.3%). EBV prevalence in colonic mucosa may contributes to high clinical disease activity in IBD patients. Takeda et al. [24] reported a UC patient with EBV detected in rectum and terminal ileum. The overall clinical picture in this patient was compatible with UC. With standard treatment for UC, his condition improved and the colonoscopy revealed improvement. It is hard to determine the presence of EBV is a bystander or an accelerator in the pathogenesis of IBD. More prospective studies are needed to explore the role of EBV in IBD.

It’s reported that long-term administration of corticosteroids and immunosuppressant therapy may activate EBV reactivation [25]. For the patients under treatment of corticosteroids, immunosuppressant or biologics, we monitor EBV activity regularly. However, there is no internationally accepted standard about the testing method, interval or the critical value. We had a patient once tested 100 times higher of EBV-DNA in his peripheral blood after 2 months biologics therapy. The patient suspended biologics and turned to mesalazine, but his symptoms relapsed. As the level of EBV-DNA decreased, he restarted the biologics with caution after 6 months rest. The level of EBV-DNA remained stable and symptoms relieved as well. Although we all know monitoring is important, it is still hard to tell on what point to terminate the therapy for patients’ best interest.

## Conclusions

Chronic active Epstein-Barr virus infection involving gastrointestinal tract is rare and very difficult to differentiate from IBD due to overlapping symptoms and endoscopic findings. Our study illustrates the need to maintain high suspicion for CAEBV among patients with intermittent febrile illnesses, extremely high level of ferritin and atypical endoscopic findings. Blood test for EBV-DNA and repeated biopsy for EBER by in situ hybridization should be performed to gain more information to confirm the diagnosis.

## Data Availability

The datasets used and/or analyzed during the current study are available from the corresponding author on reasonable request.

## Abbreviations

CAEBV: Chronic active Epstein-Barr virus infection
IBD: Inflammatory bowel disease
EBV: Epstein-Barr virus
EBER: EBV-encoded small nuclear RNA
ISH: In situ hybridization
CD: Crohn disease
UC: Ulcerative colitis
ESR: Erythrocyte sedimentation rate
CRP: C-reactive protein
PBMC: Peripheral blood mononuclear cell
